# Shedding Light on Physical Fitness During Childhood: Insights From Japan's Fitness Survey

**DOI:** 10.1111/ppe.13158

**Published:** 2025-01-07

**Authors:** Tomoko Aoyama, Naho Morisaki

**Affiliations:** ^1^ Department of Social Medicine National Center for Child Health and Development Tokyo Japan

In this issue of *Paediatric and Perinatal Epidemiology*, Yoshikawa and colleagues [1] explore the relationship between lifestyle factors and physical fitness among children, using data collected from 12 public primary schools in the northern Kanto region of Japan. This commentary aims to clarify the role of physical fitness in public health and to provide insights into future directions for surveying, monitoring, and researching fitness during childhood.

With increasing evidence that physical fitness is associated with a wide range of current and future health outcomes in children [2] and adults [3], there has been growing recognition that measuring youth physical fitness can be a helpful tool for monitoring the health status of children and predicting future disease burden. Physical fitness has traditionally received less attention than obesity and physical activity in discussions about children's health, lacking established guidelines or recommendations such as those for physical activity and body mass index (BMI). However, a global movement has recently emerged to focus on physical fitness during childhood, following the declines in children's physical fitness [4].

Large‐scale physical fitness surveys of children have been conducted in several major countries [5]; however, rarely has it been conducted periodically for health monitoring and surveillance. Following an international consensus on priorities for physical fitness research and surveillance among children and adolescents, led by Lang et al. [6], a globally standardised test battery and protocols for health monitoring and surveillance in children aged 6–18 was proposed [7]. To our knowledge, Japan is the only nation currently providing population‐based surveillance for children across all four components proposed in the globally standardised test battery [8, 9]: the 20 m shuttle run (for cardiorespiratory fitness [CRF]), handgrip strength, standing long jump (for muscular fitness) and BMI (as a proxy of body size/composition). This recent international consensus [6, 7] may encourage more countries to implement a physical fitness survey based on a globally standardised test battery.

The study by Yoshikawa et al. [1] is unique in utilising data from Japan's fitness survey for public health research, as this data have primarily been used for individual schools to understand students' physical fitness and health and to plan and implement school‐based programs for effective health and physical education. Yoshikawa et al. provided a comprehensive approach by examining how various lifestyle factors, including diet, exercise and sleep, contribute to physical fitness. Japan's physical fitness survey for youth, officially known as the ‘National Survey of Physical Fitness, Athletic Performance and Exercise Habits’ [8] (hereafter referred to as the ‘JP Fit Survey for Youth’), was initiated in 2008 and has since targeted all students in the 5th (ages 10–11) and 8th (ages 13–14) grades. Approximately 2 million students participate each year [8].

Interestingly, most of the associations they found were on the 20 m shuttle run [1], a test of CRF. Among various fitness components, CRF has been identified as the strongest predictor of future health among components of physical fitness. There is extensive research linking it to mortality [3] and overall health outcomes, including obesity, cardiovascular disease risk and mental health. CRF is a stronger predictor of cardiovascular disease risk than physical activity. Since CRF levels established during childhood and adolescence often persist into adulthood, ensuring adequate fitness during these developmental stages is a public health priority.

CRF has also been identified as an alternative measure for evaluating physical activity [10]. Subjective measurements of physical activity, such as self‐reports, are widespread in large‐scale studies; however, they are susceptible to recall and response biases and issues related to validity. While objective measurements of physical activity, such as accelerometers, can provide accurate data, their implementation at the population level is often constrained by high cost and time‐consuming issues. Since CRF is primarily determined by habitual physical activity, it can serve as a reliable proxy for assessing routine physical activity levels. Therefore, measuring CRF with a 20 m shuttle run may be advantageous, as it is cost‐effective and feasible for large populations.

If large population‐based data on physical fitness, including CRF, become available, it may enhance future studies in epidemiology and public health. Recent efforts can be seen in Europe, where an international initiative called FitBack (www.fitbackeurope.eu), a collaborative network aimed at supporting the monitoring of physical fitness among children and youth across Europe, has been launched. FitBack uses the ALPHA (Assessing Levels of PHysical Activity and fitness at population level) test battery, which includes the 20 m shuttle run to assess CRF for children and adolescents. This initiative emphasises the importance of objective data on physical fitness as an essential component in promoting physical literacy. Enhancing such efforts to assess children's CRF across diverse regions and countries could significantly advance epidemiological research promoting lifelong health.

A potential challenge of the study by Yoshikawa et al. [1] is that the lifestyle changes and decreased physical fitness associated with COVID‐19 make it unclear to what extent these findings apply to children in the post‐COVID era. While a global decline in physical fitness was a public concern before the pandemic, a significant decline in 20 m shuttle run performance was observed among the eight fitness components assessed in the JP Fit Survey for Youth during the pandemic [9]. This decline was attributed to limited opportunities for vigorous and continuous physical activities due to pandemic‐related restrictions, such as school and recreation facility closures and the cancellation or restriction of organised sports. The last national update from the Japan Sports Agency indicates that even after restrictions were lifted, their CRF appears to remain below pre‐COVID‐19 levels (Figure 1). It would be valuable to conduct additional analyses to examine what aspects of post‐COVID lifestyle changes contribute to the decline in CRF, and what public health efforts are required to mitigate this trend.

**FIGURE 1:**
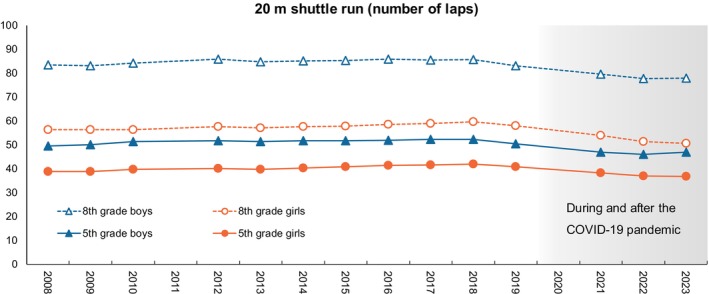
Annual trends in cardiorespiratory fitness among 5th (ages 10–11) and 8th (ages 13–14) graders in Japan from 2008 to 2023. The figure is based on the results of the JP Fit Survey for Youth [in Japanese]: https://www.mext.go.jp/sports/content/20240115‐spt_sseisaku02‐000032954_11.pdf. No nationwide survey was conducted in 2011 due to the Great East Japan Earthquake and in 2020 due to the COVID‐19 pandemic.

In summary, there is an increasing need to survey, monitor and research physical fitness to improve children's fitness levels and mitigate long‐term health risks. Specifically, there is a growing need for high‐quality research, including longitudinal studies that investigate the impact of childhood physical fitness on future health outcomes. We emphasise the need for greater attention to children's physical fitness to safeguard their lifelong health. Population‐based surveillance initiatives may provide a necessary framework for collecting longitudinal fitness data.

## Author Contributions

N.M. was asked to provide the commentary. T.A. conducted the analysis and drafted the initial version of the manuscript. N.M. critically reviewed the manuscript.

## Conflicts of Interest

The authors declare no conflicts of interest.

## Data Availability

The data described in this article are openly available online: Japan Sports Agency. Report on National Survey of Physical Fitness, Athletic Performance and Exercise Habits (FY 2023) [in Japanese]. Published December 2023. https://www.mext.go.jp/sports/b_menu/toukei/kodomo/zencyo/1411922_00007.html (Accessed November 19, 2024).
